# Lung ultrasound among Expert operator’S: ScOring and iNter-rater reliability analysis (LESSON study) a secondary COWS study analysis from ITALUS group

**DOI:** 10.1186/s44158-024-00187-x

**Published:** 2024-07-31

**Authors:** Enrico Boero, Luna Gargani, Annia Schreiber, Serena Rovida, Giampaolo Martinelli, Salvatore Maurizio Maggiore, Felice Urso, Anna Camporesi, Annarita Tullio, Fiorella Anna Lombardi, Gianmaria Cammarota, Daniele Guerino Biasucci, Elena Giovanna Bignami, Cristian Deana, Giovanni Volpicelli, Sergio Livigni, Luigi Vetrugno

**Affiliations:** 1 Department of Anaesthesia and Intensive Care Unit, San Giovanni Bosco Hospital, Turin, Italy; 2 Department of Surgical, Medical and Molecular Pathology and Critical Care Medicine, University of Pisa, Pisa, Italy; 3 Keenan Research Centre, Li Ka Shing Knowledge Institute, Unity Health Toronto (St. Michael’s Hospital), Toronto, Canada; 4 Interdepartmental Division of Critical Care Medicine, University of Toronto, Toronto, Canada; 5 Emergency Department, Barts Health NHS Trust, London, UK; 6 Saint Bartholomew’s Hospital, London, UK; 7 Department of Innovative Technologies in Medicine and Dentistry, Gabriele d’Annunzio University of Chieti-Pescara, Chieti, Italy; 8 Department of Anesthesiology, Critical Care Medicine and Emergency, SS. Annunziata Hospital, Chieti, Italy; 9 Division of Pediatric Anesthesia and Intensive Care, Buzzi Children’s Hospital, Milan, Italy; 10 Department of Medicine, University of Udine, Udine, Italy; 11 Institute of Clinical Physiology, National Research Council, Lecce, Italy; 12 Department of Translational Medicine, Università del Piemonte Orientale, Novara, Italy; 13 Department of Clinical Science and Translational Medicine, Tor Vergata’ University of Rome, Rome, Italy; 14 Anesthesiology, Critical Care and Pain Medicine Division, Department of Medicine and Surgery, University of Parma, Parma, Italy; 15 Department of Anaesthesia and Intensive Care, Health Integrated Agency of Friuli Centrale, Udine, Italy; 16 Department of Medical and Surgical Science, Magna Graecia University, Catanzaro, Italy; 17 Department of Medical, Oral and Biotechnological Sciences, University of Chieti-Pescara, Via Dei Vestini N 33, Chieti, 66100 Italy

**Keywords:** Lung sonography, Inter-rater variability, Lung ultrasound score, Intensive care

## Abstract

**Background:**

Lung ultrasonography (LUS) is a non-invasive imaging method used to diagnose and monitor conditions such as pulmonary edema, pneumonia, and pneumothorax. It is precious where other imaging techniques like CT scan or chest X-rays are of limited access, especially in low- and middle-income countries with reduced resources. Furthermore, LUS reduces radiation exposure and its related blood cancer adverse events, which is particularly relevant in children and young subjects. The score obtained with LUS allows semi-quantification of regional loss of aeration, and it can provide a valuable and reliable assessment of the severity of most respiratory diseases. However, inter-observer reliability of the score has never been systematically assessed. This study aims to assess experienced LUS operators’ agreement on a sample of video clips showing predefined findings.

**Methods:**

Twenty-five anonymized video clips comprehensively depicting the different values of LUS score were shown to renowned LUS experts blinded to patients’ clinical data and the study’s aims using an online form. Clips were acquired from five different ultrasound machines. Fleiss-Cohen weighted kappa was used to evaluate experts’ agreement.

**Results:**

Over a period of 3 months, 20 experienced operators completed the assessment. Most worked in the ICU (10), ED (6), HDU (2), cardiology ward (1), or obstetric/gynecology department (1). The proportional LUS score mean was 15.3 (SD 1.6). Inter-rater agreement varied: 6 clips had full agreement, 3 had 19 out of 20 raters agreeing, and 3 had 18 agreeing, while the remaining 13 had 17 or fewer people agreeing on the assigned score. Scores 0 and score 3 were more reproducible than scores 1 and 2. Fleiss’ Kappa for overall answers was 0.87 (95% CI 0.815–0.931, *p* < 0.001).

**Conclusions:**

The inter-rater agreement between experienced LUS operators is very high, although not perfect. The strong agreement and the small variance enable us to say that a 20% tolerance around a measured value of a LUS score is a reliable estimate of the patient's true LUS score, resulting in reduced variability in score interpretation and greater confidence in its clinical use.

**Supplementary Information:**

The online version contains supplementary material available at 10.1186/s44158-024-00187-x.

## Background

Lung ultrasonography (LUS) is a non-invasive imaging technology used in medical practice to diagnose and monitor a variety of conditions, including acute pulmonary edema, acute respiratory distress syndrome (ARDS), pneumonia, pneumonitis, atelectasis, pleural effusion, and pneumothorax [1]. It is beneficial in settings where other imaging techniques, such as computed tomography (CT) scans or chest X-rays, may not be readily available or feasible, such as in low- and middle-income countries and in resource-constrained settings of high-income ones [2]. Additionally, LUS represents a safer alternative to other imaging modalities in intensive care, reducing the exposition to ionizing radiation, especially in pediatric populations or pregnant women [3, 4]. LUS has shown to be able to provide a semi-quantitative assessment of disease severity [5]. In fact, by analyzing the lung surface over 12 thoracic zones, a clinically useful score can be obtained [6]. This score can be used to evaluate the re-aeration or de-aeration during respiratory diseases and the prognosis of COVID-19 patients with interstitial pneumonia, where higher scores suggest worse outcomes and the need for invasive mechanical ventilation [7]. In contrast, lower scores suggest a better prognosis and less invasive support [8]. In other words, LUS has been recently described and used to evaluate the underlying disease trajectory in vast number of cardiopulmonary conditions [9, 10]. In the neonatology and pediatric setting, it has been used to evaluate the need for surfactant [11], bronchopulmonary dysplasia development [12], bronchiolitis, and the need for mechanical ventilation [13]. Lung ultrasound has also been applied in the weaning phase from mechanical ventilation to predict success or failure [14], and prognostic evaluation of different conditions such as onchoemathologic diseases [15], head and neck surgery [16], hip fracture complications [17], ARDS diagnosis, and mechanical power relationship [18]. As a repeatable technique, LUS monitoring role has emerged soon, and it has been applied to monitoring disease evolution in both classic and COVID-19-related ARDS [19], both in adults and children [20]. It has also been applied to evaluate the effectiveness of pharmacological therapy and ventilation settings [6]. On the cardiovascular side, applications of LUS are well described in terms of evaluation of extra-vascular lung water [21], differential diagnosis of acute decompensated heart failure [22], and prognostic evaluation of surgical patients [23]. Its established role solicited the effort of creating an automated quantitative analysis and of using a remotely controlled robot to perform LUS [24]. Collecting more and more evidence about condition-specific cutoffs, quantitative thresholds of LUS findings have been proposed for some of these applications. The benefit would be to allow clinicians to use LUS as a diagnostic test with a dichotomous outcome, such as normal and abnormal, or high-risk and low-risk, with different actions following different results. Among LUS findings, the LUS score seems to be the most adaptable for quantitative use. However, the problem of inter-rater reliability remains. In fact, in ultrasound imaging, one of the main limitations is the dependence on the operator, both in technical expertise and in the interpretation of findings. These are crucial factors in the accuracy of ultrasound diagnosis [25]. While there is a consensus on the minimum requirements for an inexperienced operator to acquire competence, to what extent the agreement among expert operators reduces misinterpretations of abnormal findings has yet to be discovered. Therefore, this study aims to evaluate the inter-rater reliability of experienced LUS operators when assessing a predefined set of LUS findings.

## Methods

### Study design

This observational agreement study was a secondary analysis of the COWS study performed at the San Giovanni Bosco Hospital, Turin, Italy (ID protocol #82,995) [8]. Of these, patients give their permission for image and clip use. We used 25 anonymized video clips that respected the European General Data Protection Regulation 2016/679 (GDPR) and attached to this research as supplemental material. The study was conducted in accordance with the Declaration of Helsinki (as revised in 2013). We focalized on LUS and excluded critical care echocardiography, abdominal, vascular, and other point-of-care ultrasound applications. The accuracy prognostic score based on LUS to predict critical illness was assessed.

### Panel selection

Participants had to be recognized LUS experts with different expertise (10 in the emergency medicine and 10 in intensive care setting) with at least 10 years of experience in daily LUS practice. Additionally, LUS teaching experience or direct involvement in LUS research was required. Two authors (EB and LV) contacted every panel member and proposed to participate in this investigation. To balance the expert panel and the results interpretation, 3 non-experts in anesthesia and intensive care in managing the experiment were included. The lung ultrasound experts gave their approval, were unaware of the research’s objective, and could start the video clip evaluation at any time. Parameters that were recorded and scored after obtaining their consent were anonymously collected.

### LUS score calculation

The lung ultrasound score is typically calculated by dividing the lungs into 12 areas, six on each side of the chest, and each area was evaluated for the presence of 4 different lung aeration patterns. This first grade (score 0) corresponds to the absence of B-lines or their presence to a maximum of two within the worst scan of the single area. The second and third grade corresponds to the presence of B-lines, ranging from a minimum of three to a condition of coalescent B-lines. If the B-lines occupy less than or equal to 50% of the pleural line, the area is assigned a score of 1, otherwise a score of 2. The last grade of severity (score 3) is determined by any subpleural consolidation with at least 10 mm of length at the pleural level, without further differentiation between small and large consolidations. Multiple variations to this score have been proposed, but the authors decided to keep the reproducibility analysis focused on this definition. Twenty-five video clips from a total of 21 patients were included in the pool. Video clips were selected to homogenously cover the different levels of severity of the LUS score. The standard assignment of the scores was initially evaluated by two authors (EB, LG), and the discrepancies were resolved by a third (LV). After selection, 7 video clips were included in the test with a preassigned score of 0 and 6 for every preassigned score of 1, 2, and 3. In light of this, the total LUS score assigned to the 25 video clips of the test was 36, but participants’ individual answers could theoretically span from a minimum of 0 to a maximum of 75. After participants completed the test, each individual result was scaled to 36 (the scale used in clinical reality, ranging from 0 to 36) to obtain a proportional LUS (pLUS).

### Clip selection and online test

Each video clip had to be acquired using low-frequency curvilinear probes, with internal frequency at the maximum range, depth 10 cm ± 2 cm, and focus at the level of the pleural line ± 2 cm according to the standard execution of LUS [26]. All video clips were recorded for 4 to 6 s. To balance the contribution from multiple ultrasound machines, we asked to score 25 equally distributed clips among five different models (5 video clips per machine) that were chosen according to a local availability (Esaote MyLab 7®, GE LogiQ®, Butterfly iQ®, Sonosite M-Turbo®, and Philips SparQ®). All video clips are available as Supplementary materials.

The online test was built using Google Forms® through a multiple-choice quiz. Each video clip was shown without clinical or technical details. The responders could rate each video clip with a single score between 0 and 3. All answers were mandatory, and no corrections, tips, or feedbacks were given during or at the end of the test. Videos were presented one by one in a random, software-generated sequence.

### Power analysis

Given the study’s design, and in the absence of robust priors for sample size calculation, we planned to enrol an arbitrary number of 20 experts, which is an assumably adequate sample to draw significant conclusions on these specific endpoints [27].

### Statistical analysis

Continuous variables were expressed as mean values (± standard deviation) or median values with interquartile ranges (IQR) according to their distribution (Shapiro–Wilk test). Discrete variables were expressed as numbers and percentage values. In our analyses, we performed weighted kappa since using weighting schemes allows us to consider the closeness of agreement between categories. To compute weighted kappa, we used the Fleiss-Cohen weights based on inverse-square spacing. The Fleiss-Cohen system is also known as quadratic weights because it is proportional to the square of the deviation of separate ratings. In our case, with four levels, the weights used have been 1, 0.89, 0.55, and 0 for differences of 0, 1, 2, and 3, respectively.

## Results

From May to July 2020, 25 experienced operators were invited to participate in the study. Of these, 20 completed the video clip assessment on time. Fourteen males (70%) and six females were involved, with a mean age of 41.8 years (SD 8.2 years). Ten respondents worked predominantly in the ICU, 6 in the emergency department (ED), 2 in high-dependency units (HDU), 1 in the cardiology ward, and 1 in the obstetric/gynecology department (Table 1).

**Table 1:** Characteristics of video clips and evaluators. *ICU* intensive care unit, *ED* emergency department, *HDU* high-dependency unit

**Evaluators**	
Gender, *N* (%)	
Female	6 (30)
Male	14 (70)
Not declared	0 (0)
Age, mean (SD)	41.8 (8.2)
Main specialty of clinical practice, *N* (%)	
ICU	10 (50)
ED	6 (30)
HDU	2 (10)
Cardiology	1 (5)
Obstetrics/gynecology	1 (5)
Video clips	
Machine	
Esaote MyLab 7	1
GE LogiQ	1
Butterfly iQ	1
Sonosite M-Turbo	1
Philips SparQ	1
Preassigned score, *N* (%)	
Score 0	7 (28.0)
Score 1	6 (24.0)
Score 2	6 (24.0)
Score 3	6 (24.0)

**Fig. 1:**
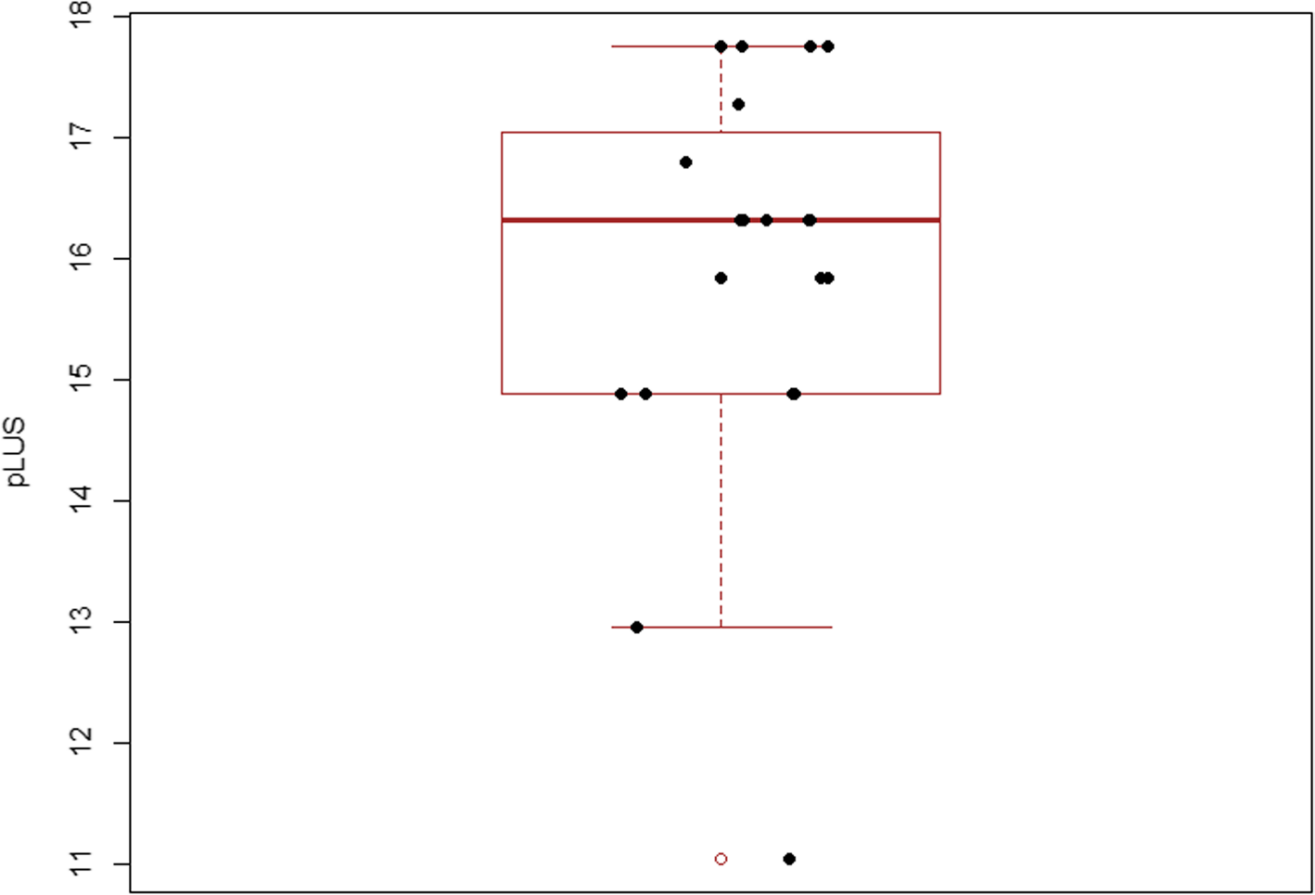
Box-plot of observed distribution of proportional LUS (pLUS) score (the empty dot indicates an outlier)

**Fig. 2:**
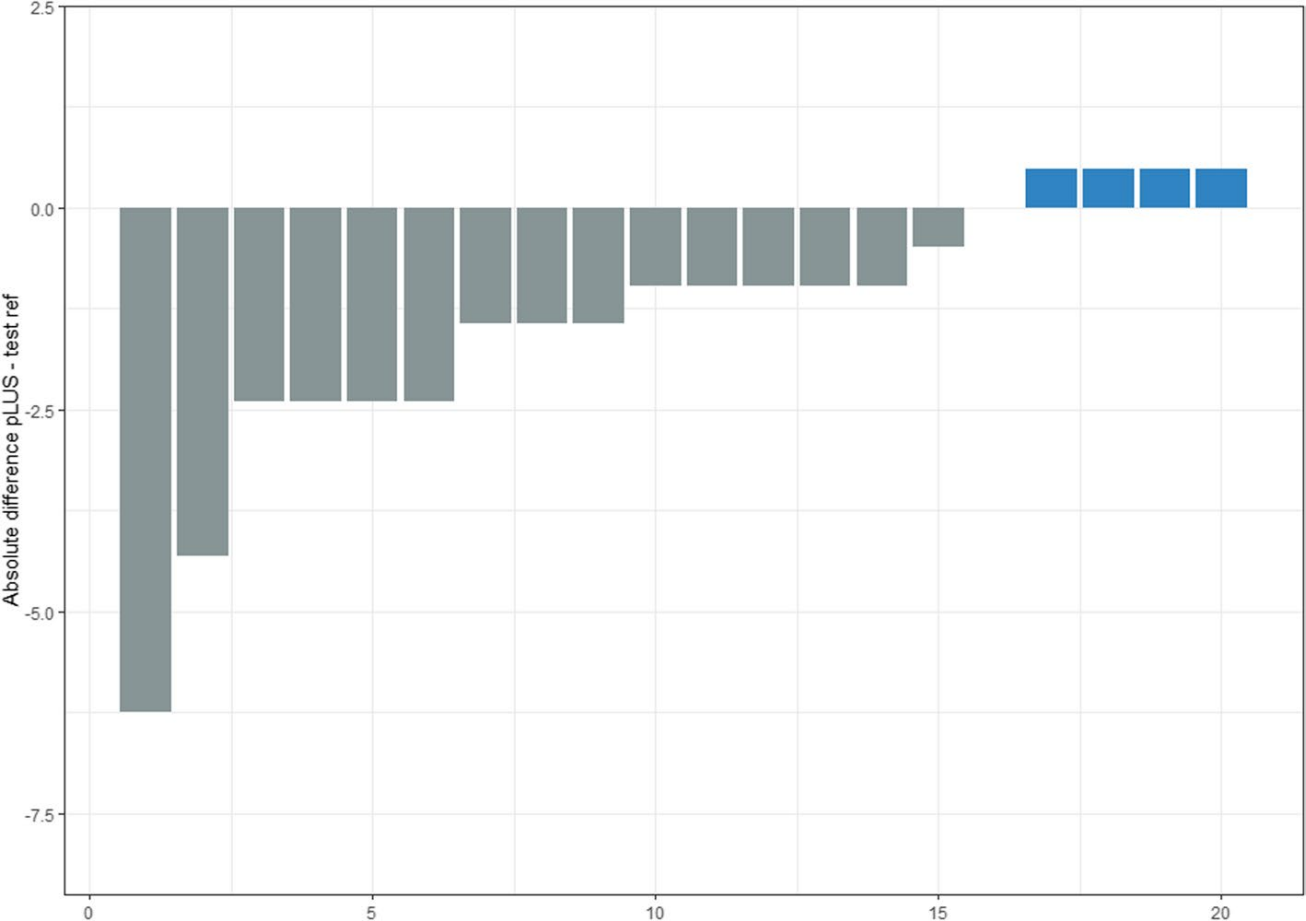
Absolute difference of proportional LUS between each rater and the test reference in crescent order

**Fig. 3:**
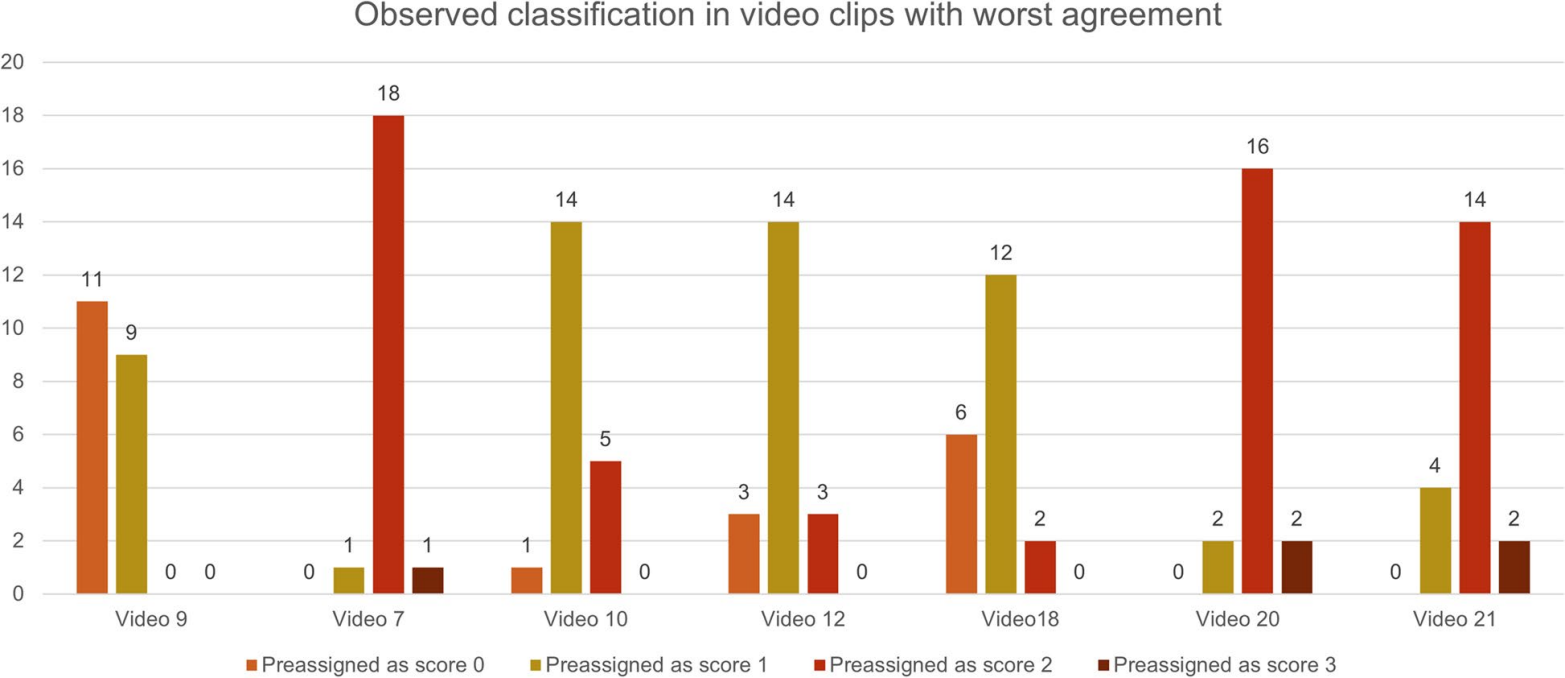
Absolute frequency of the evaluated score of the video clips with worst agreement. Video 9 showed a bimodal classification, while the other showed a normal distribution around score 1 and score 2

**Fig. 4:**
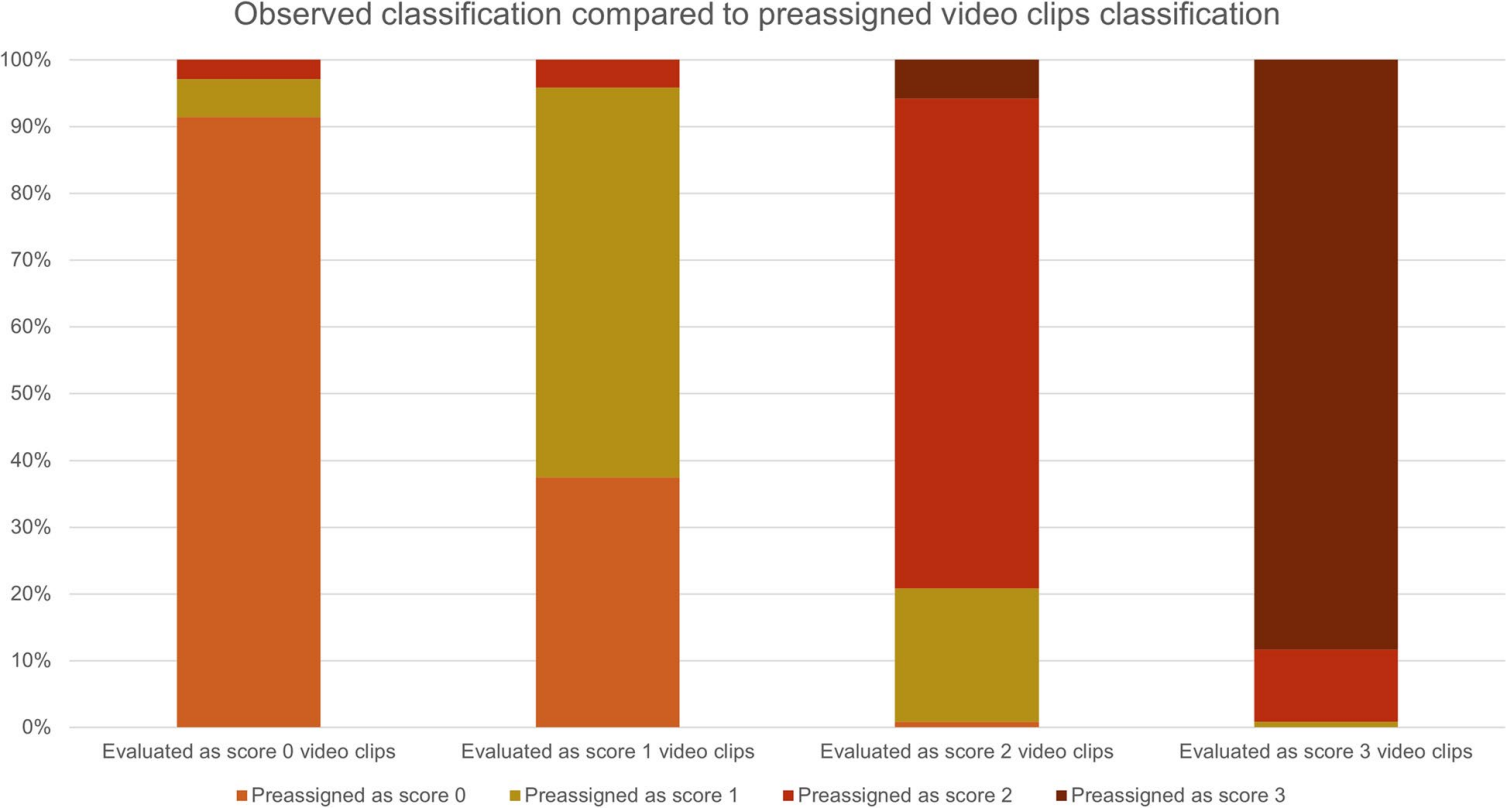
Relative frequency of the evaluated different scores among the four classes of video clips

Our sample’s median total LUS score was 33, with an interquartile range (IQR) between 31 and 35.5 (Fig. 1). The mean proportional LUS score was 15.3 (median 15.7, IQR 14.3–16.4). As the pLUS of the test would be 17.28, the difference in each rater’s pLUS from this reference has ranged from − 6.24 to + 0.48, with most of the values within ± 2 from the reference (Fig. 2). Among the set of 25 video clips, 6 of them had a full agreement, with all the 20 raters providing the same answer. Three video clips showed the agreement of 19 raters out of 20; 3 had 18 raters giving the same answer, and 1 had 17 raters agreeing. Of the remaining video clips, in 12 the agreeing raters count ranged between 12 and 16. Six of these have been assigned three different scores, showing a normal distribution around the modal value (i.e., the modal value of LUS was score 1 or 2). Only one case showed a bimodal result of 11 raters providing a score of 1, in contrast to the other 9 providing a score of 0 (Fig. 3). Among the 6 video clips originally rated as score 3 by the authors, they have been correctly classified 106 times out of 120 evaluation (88.3%). Similarly, the 7 video clips planned to represent the score 0 were correctly rated 128 times out of 140 (91.4%). On the opposite, scores 1 and 2 were correctly classified 58.3% and 73.3% of the time, respectively. Three score-1 and one case of score-2 video clips were rated mostly one class less than initially intended. None of the video clips got all four possible ratings (Fig. 4). Eighty-two non-modal ratings were registered one class away from the most-rated score, and only five observations were registered two classes away from the most-rated one. Evaluating the overall sample of answers, the quadratic weighted Fleiss’ Kappa was 0.87326 (95% CI 0.815–0.931, *p*-value < 0.001).

## Discussion

Our work is the first focusing on inter-rater evaluation of LUS scores among experienced physicians. Its focus on video clips of conditions that have been evaluated before COVID-19 allows us to consider the accuracy of the LUS score system on the common ED and ICU patients without possible biases due to the exceptional increase in interest in LUS during the pandemic. We observed a strong agreement between operators, with a kappa of 0.87, which allows us to state that, given a reasonable amount of training in LUS, this measure might not be as operator-dependent as previously stated. In particular, extreme scores, such as 0 and 3, more relevant from a clinical standpoint, showed better agreement. In contrast, correctly classifying low LUS score patients is of utmost importance since the LUS score is mostly useful to individuate low-risk cases. In fact, relevant literature shows that the LUS score brings a consistently high negative predictive value when used for prognostic purposes [28]. On the other hand, while an increase in LUS score reflects a proportional increase in lung density, only a score of 3 indicates a complete loss of aeration and therefore has relevant consequences in terms of pulmonary shunt and functional lung impairment [29].

Video clips with findings preassigned as scores 1 and 2 showed lesser concordance, which may be due to several reasons and some of them are intrinsic limits of this study. First, video clips with borderline cases were actively sought (i.e., video clips showing exactly 3 B-lines, or B-lines covering nearly 50% of the pleural line) to be consistent with real-world scenarios, where some intermediate findings are common experience. This may have widened the spread of answers around scores 1 and 2. Second, we could not control how the participants took the test, in particular in what conditions of lighting and on what kind of devices (e.g., personal computers, tablets, or mobile). This might have introduced more variation, particularly in identifying B-lines and eyeballing the dimensions of small subpleural consolidations. Lastly, we did not provide the experienced operators who took part in the study with the definition of the LUS score used for this test, which was well-endorsed by the authors. Therefore, we could only assume that the LUS scores used were consistent with each other.

We recognized a slight decrease in the median assigned LUS score in comparison to the expected from the author’s video clip selection. The latter should have been 36 due to the homogeneous distribution of video clips among the four classes of the LUS score. However, the observed median has been 33. This is mostly driven by four video clips (video 9, video 10, video 16, video 17) that have consistently been underrated by 1 point (i.e., from score 2 to 1 or from 1 to 0). On the opposite, clips numbered 7, 8, 20, and 21 were all correctly rated as score 2 by the most, but some classified them as score 3, possibly for the presence of an irregular pleural line. The reasons for these inconsistencies might be found in the ones mentioned above, with particular regard to the device used.

Whether the video clips were seen on small screens or big high-quality monitors, some intrinsic limitations remain in the human interpretation of them. The LUS score is a semi-quantitative method for the assessment of lung aeration and de-aeration, which works well in the extreme score 0 (totally aerated) and score 3 (totally de-aerated), but only moderately well in the middle scores (2 and 3). In a similar field of respiratory imaging, artificial intelligence (AI) and automated algorithms have recently been used to overcome the well-known limitations of human evaluation of diagnostic imaging. This might help the physicians uniquely score the images they view. For example, an algorithm for systematic, objective fibrotic imaging analysis (SOFIA) was tested by Walsh et al. against the radiologist’s usual interstitial pneumonia (UIP) probability [30]. In this case, only SOFIA predicted survival when prognostic accuracy in the detection of the UIP pattern was assessed. Furukawa et al. have been tested an AI algorithm to evaluate the diagnostic accuracy of the diagnosis of idiopathic pulmonary fibrosis (IPF), when clinical data were additionally incorporated into the assessment [31]. Moreover, different software has been used to evaluate the diagnostic accuracy in the screening of pulmonary tuberculosis, resulting in high sensitivity for the AI identification of the illness. In Marozzi et al., an automatic algorithm has been tested to support non-expert physicians in interstitial pneumonia evaluation [32]. The study reports that the algorithm provides a quantitative score for each analyzed patient non-inferior to expert physicians. Similar results have been obtained in Lombardi et al., where a high agreement between the algorithm and the expert operator evaluations was observed [33]. As far as concern LUS score evaluations, there are no studies available so far that take into account the use of AI, but it is reasonable to think that similar improvements can be brought into the clinical scenario by this currently evolving innovation also for the LUS score calculation.

This study has been carried out on a small sample of very well-prepared operators, but the casual error may still have played a role, and a larger repetition of this investigation is needed to provide definitive data. In particular, testing a sample of video clips with a subset of predefined borderline and non-borderline findings would be interesting. Even if efforts have been made to cover a wide variety of them, the extension to more ultrasound machines may provide more real-life insights. Although it is impossible to exclude that our results are influenced by previous operator’s clinical expertise, limited data compares results based on different expertise in different clinical practices. The hidden profile paradigm, that occurs in the process of group decision making, could be present in our study in one or more operators that poorly classified the LUS score due to unshared information [34].

Our study may have implications for clinical practice, research, and teaching. First, one may wonder what the real meaning of a registered value of LUS score on a medical chart is. Is that a precise amount that one can use to risk stratify patients and predict disease trajectories in ED and ICU? The answer to this question may be clearer now, considering that our results showed a mean LUS score of 15.3, but, mostly, a standard deviation of 1.6, and this second value might be the most interesting. An SD of 1.6 out of a mean of 15.3 would mean that a 10% tolerance on a patient’s given score might contain, with 68% probability, the real value of the patient’s LUS score. Allowing for a larger 20% tolerance, it may include 95% of possible real values in a specific patient. Bringing this to a real-life scenario means that finding a LUS score of 10 would be a reliable (95%) estimate of a true LUS score between 8 and 12 [35]. Second, some studies provide similar but conflicting data on LUS score thresholds for various purposes [36, 37]. The need for mechanical ventilation, the prediction of ICU admission, the prediction of weaning from mechanical ventilation, the possibility to safely discharge home a patient from the ED, and the prediction of postoperative complications are just some examples [38, 39]. Many of the reported cutoffs range between 12 and 17 points of the LUS score. The inconsistencies registered among these studies may be partly explained by a 10 to 20% variability in the true value of the LUS score of included patients. Third, from a teaching point of view, it is of utmost importance to maintain a consistent way of acquiring and interpreting LUS video clips, coupled with a consistent and universal definition of the LUS score to be used in further studies [40]. Finally, a test built according to our template may be used to certify completion of training and to provide periodical follow-up among providers with a low volume of LUS cases.

## Conclusions

We investigated the inter-rater agreement between experienced LUS operators and found a strong agreement. This allows us to conclude that a registered LUS score value, associated with a 10 to 20% tolerance, is a reliable estimate of the patient’s true LUS score, when well-experienced operators are assessing it. This brings lesser variability in score interpretation and allows more confidence in the use of LUS score.

### Supplementary Information

Additional file 1: Video clips

## Data Availability

No datasets were generated or analysed during the current study.

## Abbreviations

LUS: Lung ultrasound
CT: Computed tomography
ICU: Intensive care unit
ED: Emergency department
HDU: High dependent unit
ARDS: Adult respiratory distress syndrome
GDPR: General Data Protection Regulation
SOFIA: Fibrotic imaging analysis
UIP: Usual interstitial pneumonia

## References

[1] Vetrugno L, Biasucci DG, Deana C, Spadaro S, Lombardi FA, Longhini F et al (2024) Lung ultrasound and supine chest X-ray use in modern adult intensive care: mapping 30 years of advancement (1993–2023). Ultrasound J 16(1):7. 10.1186/s13089-023-00351-4 38345653 10.1186/s13089-023-00351-4 PMC10861418

[2] Matthay MA, Arabi Y, Arroliga AC, Bernard G, Bersten AD, Brochard LJ et al (2024) New global definition of acute respiratory distress syndrome. Am J Respir Crit Care Med 209(1):37–47. 10.1164/rccm.202303-0558WS 37487152 10.1164/rccm.202303-0558WS PMC10870872

[3] Camporesi A, Vetrugno L, Morello R, De Rose C, Ferrario S, Buonsenso D (2023) Prognostic value of the area of lung involved in severe and non-severe bronchiolitis: an observational, ultrasound-based study. J Clin Med 13(1):84. 10.3390/jcm13010084 38202091 10.3390/jcm13010084 PMC10780043

[4] Arbeid E, Demi A, Brogi E, Gori E, Giusto T, Soldati G et al (2017) Lung ultrasound pattern is normal during the last gestational weeks: an observational pilot study. Gynecol Obstet Invest 82(4):398–403. 10.1159/000448140 27701165 10.1159/000448140

[5] Vetrugno L, Sala A, Orso D, Meroi F, Fabbro S, Boero E et al (2022) PINK-CO study investigators. Lung ultrasound signs and their correlation with clinical symptoms in COVID-19 pregnant women: the “PINK-CO” observational study. Front Med (Lausanne) 8:768261. 10.3389/fmed.2021.768261 35127744 10.3389/fmed.2021.768261 PMC8814327

[6] Bouhemad B, Liu ZH, Arbelot C, Zhang M, Ferarri F, Le-Guen M et al (2010) Ultrasound assessment of antibiotic-induced pulmonary reaeration in ventilator-associated pneumonia. Crit Care Med 38(1):84–92. 10.1097/CCM.0b013e3181b08cdb 19633538 10.1097/CCM.0b013e3181b08cdb

[7] Soummer A, Perbet S, Brisson S, Arbelot C, Constantin JM, Lu Q et al (2012) Lung Ultrasound Study Group. Ultrasound assessment of lung aeration loss during a successful weaning trial predicts postextubation distress*. Crit Care Med 40(7):2064–72. 10.1097/CCM.0b013e31824e68ae 22584759 10.1097/CCM.0b013e31824e68ae

[8] Boero E, Rovida S, Schreiber A, Berchialla P, Charrier L, Cravino MM et al (2021) The COVID-19 Worsening Score (COWS)-a predictive bedside tool for critical illness. Echocardiography 38(2):207–216. 10.1111/echo.14962 33491261 10.1111/echo.14962 PMC8013873

[9] Lichter Y, Topilsky Y, Taieb P, Banai A, Hochstadt A, Merdler I et al (2020) Lung ultrasound predicts clinical course and outcomes in COVID-19 patients. Intensive Care Med 46(10):1873–1883. 10.1007/s00134-020-06212-1 32860069 10.1007/s00134-020-06212-1 PMC7454549

[10] Vetrugno L, Meroi F, Orso D, D’Andrea N, Marin M, Cammarota G et al (2022) Can lung ultrasound be the ideal monitoring tool to predict the clinical outcome of mechanically ventilated COVID-19 patients? An observational study. Healthcare (Basel) 10(3):568. 10.3390/healthcare10030568 35327046 10.3390/healthcare10030568 PMC8955357

[11] De Luca D, Foti A, Alonso-Ojembarrena A, Condò V, Capasso L, Raschetti R et al (2024) UNION study group. Lung consolidations depth and gas exchange in different types of neonatal respiratory failure: the UNION multicenter study. Chest 165(6):1431–1434. 10.1016/j.chest.2024.02.012 38367957 10.1016/j.chest.2024.02.012

[12] Alonso-Ojembarrena A, Aldecoa-Bilbao V, De Luca D (2023) Imaging of bronchopulmonary dysplasia. Semin Perinatol 47(6):151812. 10.1016/j.semperi.2023.151812 37775364 10.1016/j.semperi.2023.151812

[13] Capasso L, Pacella D, Migliaro F, Salomè S, Grasso F, Corsini I et al (2023) Can lung ultrasound score accurately predict surfactant replacement? A systematic review and meta-analysis of diagnostic test studies-in reply. Pediatr Pulmonol 58(9):2685–2686. 10.1002/ppul.26558 37341615 10.1002/ppul.26558

[14] Bouhemad B, Mongodi S, Via G, Rouquette I (2015) Ultrasound for “lung monitoring” of ventilated patients. Anesthesiology 122(2):437–447. 10.1097/ALN.0000000000000558 25501898 10.1097/ALN.0000000000000558

[15] Gomez Ravetti C, Ataide TBLS, Barreto LM, Bastos FL, Gomes AGDR, Detoffol RB et al (2020) Lung ultrasound is useful in oncohematologic patients with respiratory dysfunction admitted to an intensive care unit (ICU): a pilot study. Med Ultrason 22(2):2332. 10.11152/mu-2332 32399525 10.11152/mu-2332

[16] Goel N, Sen IM, Bakshi J (2022) Lung ultrasonography as a tool to guide perioperative atelectasis treatment bundle in head and neck cancer patients undergoing free flap reconstructive surgeries: a preliminary observational study. Braz J Otorhinolaryngol 88(2):204–211. 10.1016/j.bjorl.2020.05.030 32800584 10.1016/j.bjorl.2020.05.030 PMC9422385

[17] Vetrugno L, Boero E, Bignami E, Cortegiani A, Raineri SM, Spadaro S et al (2021) LUSHIP Study Investigators. Association between preoperative evaluation with lung ultrasound and outcome in frail elderly patients undergoing orthopedic surgery for hip fractures: study protocol for an Italian multicenter observational prospective study (LUSHIP). Ultrasound J 13(1):30. 10.1186/s13089-021-00230-w 34100124 10.1186/s13089-021-00230-w PMC8184059

[18] Smit MR, Hagens LA, Heijnen NFL, Pisani L, Cherpanath TGV, Dongelmans DA et al (2023) DARTS Consortium members. Lung ultrasound prediction model for acute respiratory distress syndrome: a multicenter prospective observational study. Am J Respir Crit Care Med 207(12):1591–601. 10.1164/rccm.202210-1882OC 36790377 10.1164/rccm.202210-1882OC PMC10273105

[19] Vetrugno L, Bove T, Orso D, Barbariol F, Bassi F, Boero E et al (2020) Our Italian experience using lung ultrasound for identification, grading and serial follow-up of severity of lung involvement for management of patients with COVID-19. Echocardiography 37(4):625–627. 10.1111/echo.14664 32239532 10.1111/echo.14664 PMC7228311

[20] Buonsenso D, Morello R, Mariani F, De Rose C, Cortese R, Vetrugno L et al (2023) Role of lung ultrasound in the follow-up of children with previous SARS-CoV-2 infection: a case-control assessment of children with long COVID or fully recovered. J Clin Med 12(9):3342. 10.3390/jcm12093342 37176782 10.3390/jcm12093342 PMC10179159

[21] Volpicelli G, Skurzak S, Boero E, Carpinteri G, Tengattini M, Stefanone V et al (2014) Lung ultrasound predicts well extravascular lung water but is of limited usefulness in the prediction of wedge pressure. Anesthesiology 121(2):320–327. 10.1097/ALN.0000000000000300 24821071 10.1097/ALN.0000000000000300

[22] Gargani L, Girerd N, Platz E, Pellicori P, Stankovic I, Palazzuoli A et al (2023) This document was reviewed by members of the 2020–2022 EACVI Scientific Documents Committee. Lung ultrasound in acute and chronic heart failure: a clinical consensus statement of the European Association of Cardiovascular Imaging (EACVI). Eur Heart J Cardiovasc Imaging 24(12):1569–82. 10.1093/ehjci/jead169 37450604 10.1093/ehjci/jead169 PMC11032195

[23] Boussier J, Lemasle A, Hantala N, Scatton O, Vaillant JC, Paye F et al (2024) Lung ultrasound score on postoperative day 1 is predictive of the occurrence of pulmonary complications after major abdominal surgery: a multicenter prospective observational study. Anesthesiology 140(3):417–429. 10.1097/ALN.0000000000004855 38064713 10.1097/ALN.0000000000004855

[24] Tsumura R, Hardin JW, Bimbraw K, Grossestreuer AV, Odusanya OS, Zheng Y et al (2021) Tele-operative low-cost robotic lung ultrasound scanning platform for triage of COVID-19 patients. IEEE Robot Autom Lett 6(3):4664–4671. 10.1109/lra.2021.3068702 34532570 10.1109/lra.2021.3068702 PMC8442628

[25] Nazerian P, Volpicelli G, Vanni S, Gigli C, Betti L, Bartolucci M et al (2015) Accuracy of lung ultrasound for the diagnosis of consolidations when compared to chest computed tomography. Am J Emerg Med 33(5):620–625. 10.1016/j.ajem.2015.01.035 25758182 10.1016/j.ajem.2015.01.035

[26] Volpicelli G, Elbarbary M, Blaivas M, Lichtenstein DA, Mathis G, Kirkpatrick AW et al (2012) International Liaison Committee on Lung Ultrasound (ILC-LUS) for International Consensus Conference on Lung Ultrasound (ICC-LUS). International evidence-based recommendations for point-of-care lung ultrasound. Intensive Care Med 38(4):577–91. 10.1007/s00134-012-2513-4 22392031 10.1007/s00134-012-2513-4

[27] Rouby JJ, Arbelot C, Gao Y, Zhang M, Lv J, An Y et al (2018) APECHO Study Group. Training for lung ultrasound score measurement in critically ill patients. Am J Respir Crit Care Med 198(3):398–401. 10.1164/rccm.201802-0227LE 29557671 10.1164/rccm.201802-0227LE PMC7205011

[28] Ji L, Cao C, Gao Y, Zhang W, Xie Y, Duan Y et al (2020) Prognostic value of bedside lung ultrasound score in patients with COVID-19. Crit Care 24(1):700. 10.1186/s13054-020-03416-1 33353548 10.1186/s13054-020-03416-1 PMC7754180

[29] Yin W, Zou T, Qin Y, Yang J, Li Y, Zeng X et al (2019) Chinese Critical Ultrasound Study Group (CCUSG). Poor lung ultrasound score in shock patients admitted to the ICU is associated with worse outcome. BMC Pulm Med 19(1):1. 10.1186/s12890-018-0755-9 30606165 10.1186/s12890-018-0755-9 PMC6318853

[30] Walsh SLF, Mackintosh JA, Calandriello L, Silva M, Sverzellati N, Larici AR et al (2022) Deep learning-based outcome prediction in progressive fibrotic lung disease using high-resolution computed tomography. Am J Respir Crit Care Med 206(7):883–891. 10.1164/rccm.202112-2684OC 35696341 10.1164/rccm.202112-2684OC

[31] Furukawa T, Oyama S, Yokota H, Kondoh Y, Kataoka K, Johkoh T et al (2022) A comprehensible machine learning tool to differentially diagnose idiopathic pulmonary fibrosis from other chronic interstitial lung diseases. Respirology 27(9):739–746. 10.1111/resp.14310 35697345 10.1111/resp.14310

[32] Marozzi MS, Cicco S, Mancini F, Corvasce F, Lombardi FA, Desantis V et al (2024) A Novel automatic algorithm to support lung ultrasound non-expert physicians in interstitial pneumonia evaluation: a single-center study. Diagnostics (Basel) 14(2):155. 10.3390/diagnostics14020155 38248032 10.3390/diagnostics14020155 PMC10814651

[33] Lombardi FA, Franchini R, Morello R, Casciaro E, Ianniello S, Serra M et al (2021) A new standard scoring for interstitial pneumonia based on quantitative analysis of ultrasonographic data: a study on COVID-19 patients. Respir Med 189:106644. 10.1016/j.rmed.2021.106644 34653873 10.1016/j.rmed.2021.106644 PMC8496946

[34] Chahine S, Cristancho S, Padgett J, Lingard L (2017) How do small groups make decisions?: a theoretical framework to inform the implementation and study of clinical competency committees. Perspect Med Educ 6(3):192–198. 10.1007/s40037-017-0357-x 28534277 10.1007/s40037-017-0357-x PMC5466572

[35] Baciarello M, Bonetti A, Vetrugno L, Saturno F, Nouvenne A, Bellini V et al (2022) Is lung ultrasound score a useful tool to monitoring and handling moderate and severe COVID-19 patients in the general ward? An observational pilot study. J Clin Monit Comput 36(3):785–793. 10.1007/s10877-021-00709-w 33948780 10.1007/s10877-021-00709-w PMC8096129

[36] Zieleskiewicz L, Markarian T, Lopez A, Taguet C, Mohammedi N, Boucekine M et al (2020) AZUREA Network. Comparative study of lung ultrasound and chest computed tomography scan in the assessment of severity of confirmed COVID-19 pneumonia. Intensive Care Med 46(9):1707–713. 10.1007/s00134-020-06186-0 32728966 10.1007/s00134-020-06186-0 PMC7388119

[37] Castro-Sayat M, Colaianni-Alfonso N, Vetrugno L, Olaizola G, Benay C, Herrera F et al (2024) Lung ultrasound score predicts outcomes in patients with acute respiratory failure secondary to COVID-19 treated with non-invasive respiratory support: a prospective cohort study. Ultrasound J 16(1):20. 10.1186/s13089-024-00365-6 38457009 10.1186/s13089-024-00365-6 PMC10923765

[38] de Alencar JCG, Marchini JFM, Marino LO, da Costa Ribeiro SC, Bueno CG, da Cunha VP et al (2021) Lung ultrasound score predicts outcomes in COVID-19 patients admitted to the emergency department. Ann Intensive Care 11(1). 10.1186/s13613-020-00799-w 10.1186/s13613-020-00799-w PMC7797883 33427998

[39] Trias-Sabrià P, Molina-Molina M, Aso S, Argudo MH, Diez-Ferrer M, Sabater J et al (2021) Lung ultrasound score to predict outcomes in COVID-19. Respir Care 66(8):1263–1270. 10.4187/respcare.08648 34006594 10.4187/respcare.08648

[40] Vetrugno L, Mojoli F, Boero E, Berchialla P, Bignami EG, Orso D et al (2022) Level of diffusion and training of lung ultrasound during the COVID-19 pandemic - a national online Italian survey (ITALUS) from the lung ultrasound working group of the Italian Society of Anesthesia, Analgesia, Resuscitation, and Intensive Care (SIAARTI). Ultraschall Med 43(5):464–472. 10.1055/a-1634-4710 34734405 10.1055/a-1634-4710 PMC9534595

